# Duration of labor in consecutive deliveries: a retrospective data analysis

**DOI:** 10.1007/s00404-024-07554-7

**Published:** 2024-05-23

**Authors:** Jessica Kreienbühl, Ladina Rüegg, Dalia Balsyte, Ladina Vonzun, Nicole Ochsenbein-Kölble

**Affiliations:** 1 Department of Obstetrics, University Hospital of Zurich, Frauenklinikstrasse 10, 8091 Zurich, Switzerland; 2 University of Zurich, Rämistrasse 71, 8091 Zurich, Switzerland; 3 Department of Obstetrics, Kantonsspital Winterthur, Brauerstrasse 15, 8400 Winterthur, Switzerland; 4 Department of Obstetrics, GZO Spital Wetzikon, Spitalstrasse 66, 8620 Wetzikon, Switzerland

**Keywords:** Consecutive labor times, Duration of labor, Labor stages, Birth weight, Head circumference, Lenght of labor

## Abstract

**Purpose:**

Labor is shorter in multiparous women. However, there are no individualized data on differences in duration of labor for consecutive deliveries in the same parturient.

**Methods:**

We conducted a retrospective data analysis from 2004 to 2021 at the University Hospital of Zurich and included all women with 2 or more vaginal deliveries of a singleton child in cephalic position, between 22 and 42 weeks of gestation. Descriptive statistics were performed with SPSS version 25.0 (IBM, SPSS Inc., USA). The primary endpoint was the ratio between durations of labor stages in consecutive deliveries of the same parturient.

**Results:**

A total of 3344 women with 7066 births (2601 first [P0], 2987 s [P1], 1176 third [P2], and 302 fourth [P3]) were included. The ratio of duration of the active first stage of labor between P1 and P0 was 0.49 (95% CI 0.47–0.51, *p* < 0.001) meaning that the active first stage of labor was 51% shorter. The second stage of labor with a ratio of 0.26 (95% CI 0.24–0.27, *p* < 0.001) was 74% shorter in P1 compared to P0. Higher birthweight of the first child led to an even greater decrease in duration of the second stage of labor in P1 compared to P0 (*p* = 0.003). Neuraxial anesthesia was an independent risk factor for a longer duration of labor, irrespective of parity (*p* < 0.001). Birthweight and HC of the neonates did not significantly differ between the children born by the same women. However, higher birthweight in of the first child significantly augmented the rate of second stage of labor between P0 and P1 (*p* = 0.003).

**Discussion:**

Up to the third delivery, duration of labor decreased with each consecutive delivery of the same parturient. An individualized assessment of the expected duration of labor in multiparous women should be encouraged.

## What does this study add to the clinical work

**Table:** 

Up to the third birth duration of labour significantly decreases (50% regarding the active first stage, 75% regarding the second stage comparing the second to the first birth) with each consecutive delivery of one parturient. With our data, duration of labour in a consecutive delivery can be estimated and can be helpful for an individualized counselling of multiparous women.	

## Introduction

Duration of labor is highly variable among women. Back in the mid-1950s, a widely accepted labor curves were published by Emanuel Friedman [1–3]. Based on the labor course of 500 nulliparous and 500 multiparous women, the minimum rate of normal cervical dilation during active phase was 1.2 cm/h and 1.5 cm/h respectively [1]. Additionally, Friedman postulated a linear progression curve of 1 cm/h and defined the beginning of active phase at 3–4 cm [1]. Since then, multiple studies have challenged the Friedman curve and found that progression of labor can be slower and non-linear with a range for nulliparous (0.29–1.57 cm/h), and multiparous (0.32–4.47 cm/h) women [4–6]. This wide variability of labor progression is most likely explained by an inter-individual variation as well as other potential factors, such as the neonatal birthweight or head circumference. Burke et al. [7], for example, found that fetal head circumference ≥ 37 cm is associated with prolonged first and second stages of labor.

In general, labor is shorter in multiparous women [1–3]. However, there are no reliable data comparing the length of labor of consecutive deliveries of the same parturient. We hypothesized that labor in a second delivery is half as long as in a fist.

Therefore, the primary aim of this study was to assess the duration of different stages of labor in subsequent deliveries of one same parturient. Secondary purpose was to evaluate the impact of additional factors, such as neonatal birthweight and head circumference on the duration of consecutive deliveries.

## Methods

This was a retrospective single-center cohort study. Data were retrieved from our electronic patient chart system at our center from 2004 to 2021. All women with two or more consecutive vaginal deliveries of a singleton child in cephalic position, and between 22 and 42 gestational weeks (GW) were included. Breech presentation, malformations, and multiple pregnancies were excluded. Additionally, cases with incomplete information about the stages of labor were excluded.

### Definitions

Stages of labor were defined as follows: the first stage of labor consists of the latent first stage (subjective start of contractions until dilation of 4 cm) and the active first stage (dilation of 4 cm until full cervical dilation); the second stage of labor defined as the time between full cervical dilation until delivery. The placental stage begins after delivery of the neonate and ends with the expulsion of the placenta.

**Fig. 1:**
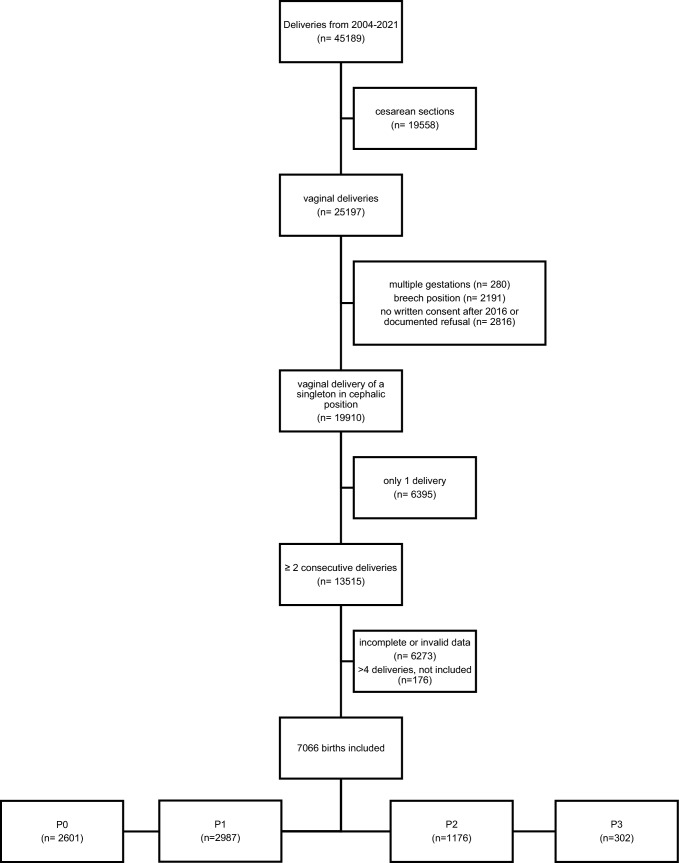
From a total of 45189 births between 2004 and 2021, 13515 were consecutive vaginal deliveries. We included a total of 7066 births

Nulliparous (P0) was defined as women that never had a vaginal delivery after 22 weeks of gestation, whereas multiparous women were defined as having had one (P1) or more vaginal deliveries (P2, P3, respectively) after 22 weeks of gestation (Fig. 1).

### Data analysis

Descriptive statistics were performed with SPSS version 25.0 (IBM, SPSS Inc., USA).

**Fig. 2:**
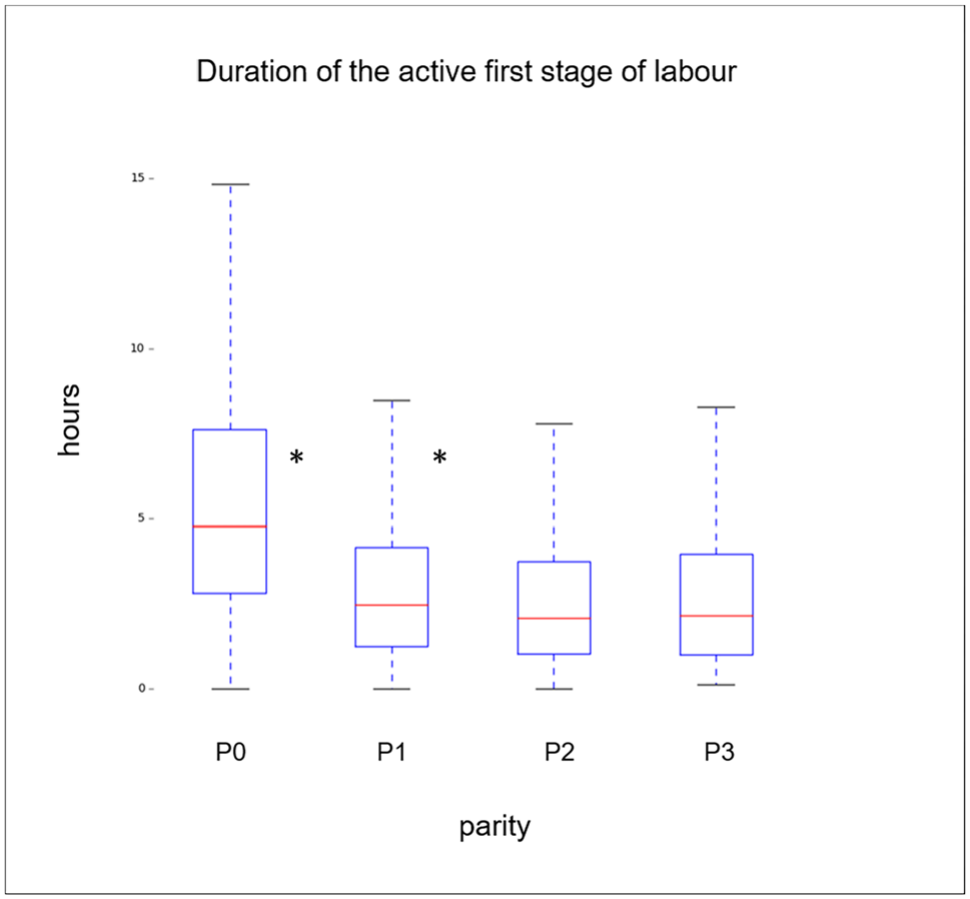
Overview of the duration of the active first stage of labor of the different parities, showing the significant (*) shortening in duration (hours) for the first two consecutive deliveries (P0 and P1 as well as P1 and P2), whereas no difference appears between the third (P2) and fourth (P3) delivery

Quantitative data are presented as mean ± standard deviation or median (IQR) depending on the distribution of data. Data comparison was done using Mann–Whitney *U* test. To compare the timing of the labor stages between nulli- and multiparous women individually, a ratio analysis was performed. The data were then presented as median and its 95% confidence interval. Statistical significance was given with *p* < 0.05. Univariate and multiple logistic regression analyses were performed to identify influencing factors on the difference of duration of the first and the second stage of labor between nulliparous and multiparous women (Fig. 2).

## Ethics approval

**Fig. 3:**
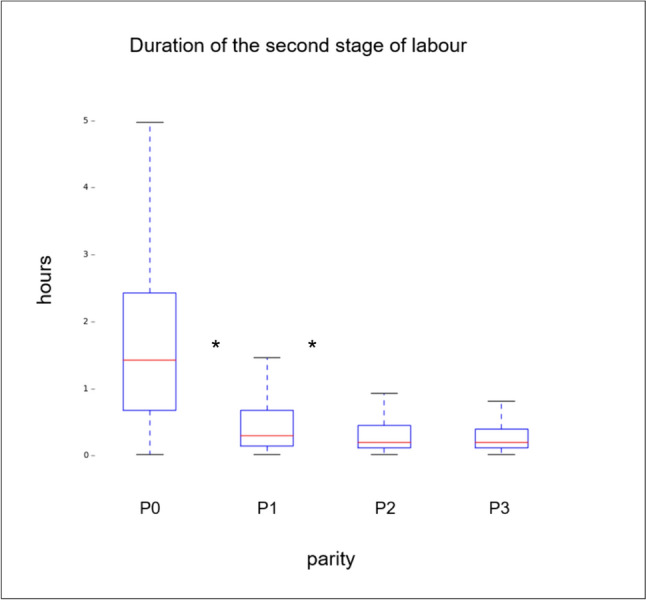
Overview of the duration of the second stage of labor of the different parities, showing the significant (*) shortening in duration (hours) for the first two consecutive deliveries (P0 and P1 as well as P1 and P2), whereas no difference appears between the third (P2) and the fourth (P3) delivery

This study was conducted with the principles of the Declaration of Helsinki and International Conference on Harmonization E6 (Good Clinical Practice) guidelines. Written informed consent was obtained from all women after 2016 when written consent was introduced at our center. Before 2016, all women were included unless written refusal was documented. The study was approved by the Ethical Committee of Zurich (KEK-ZH Nr. 2021–01632) (Fig. 3).

## Results

Of a total of 45,189 births between 2004 and 2021, 13,515 consecutive vaginal deliveries (singleton in cephalic position) were identified. After a quality control of the data, 6273 births had to be excluded due to missing or invalid data. A total of 3344 women with an overall of 7066 births were included in this study. 2601 were the first births (P0), while 2987 were the second (P1), 1176 were the third (P2), and 302 were the fourth birth. 176 births were more than the fourth delivery of the parturient and not included in the analysis. Details are shown in Fig. 1.

Baseline demographics and characteristics of the study population are also shown in Table 1. The baseline characteristics were comparable in nulli- and multiparous women. Naturally given, multiparous women were older (*p* < 0.001). Body mass index (BMI) was higher before the second and the third pregnancy (*p* < 0.001). However, at delivery, BMI was comparable (*p* = 0.54). No statistical difference in gestational age at delivery between consecutive deliveries was observed (*p* = 0.72).

**Table 1:** Baseline characteristics of the study cohort. Data of nulliparous (P0), primiparous (P1) and women that gave birth twice before (P2) are presented as median and interquartile range (IQR)

Maternal characteristics	P0 *N* = 2601	P1 *N* = 2987	P2 *N* = 1176	*p* value
Maternal age [years]; median (IQR)	30 (25–33)	32 (28–36)	33 (29–36)	< 0.001
Body mass index before pregnancy [kg/m^2^]; median (IQR)	21 (20–24)	22 (20–25)	23 (21–26)	< 0.001
Body mass index at delivery [kg/m^2^]; median (IQR)	27 (25–29)	27 (25–30)	28 (25–31)	0.54
GA at delivery, [weeks]; median (IQR)	39 (38–40)	39 (39–40)	39 (38–40)	0.72
Ethnicity, *N* (%)				
Caucasian	2311 (69%)			
Mediterranean	273 (8%)			
Asian	155 (5%)			
Orient	198 (6%)			
Afro-Caribbean	260 (8%)			
Mixed	38 (1%)			
Unknown	108 (3%)			

### Primary endpoint

The active first stage of labor was almost twice as long during the first (4 h 48 min) compared to the second (2 h 25 min) delivery (median ratio 0.49, *p* < 0.001). The second stage of labor was 74% shorter (median ratio 0.26, *p* < 0.001) comparing the second (18 min) to the first (1 h 26 min) delivery. The duration of the placental stage was identical (9 min) (median ratio 1.0, *p* = 0.06) (Table 2).

**Table 2:** Duration of different stages of labor in nulliparous (P0) and primiparous (P1). Values are presented in hours and minutes (hh:mm), median and interquartile range (IQR) as well as the ratio of the medians with a confidential interval (CI) of 95%

	P0 *N* = 2601	P1 *N* = 2987	*p* value	Ratio P1:P0 (95% CI)
Active first stage of labor, [hh:mm], median (IQR)	04:48 (2:50–07:40)	02:25 (01:15–04:05)	** < 0.001**	**0.49** **(0.47–0.51)**
Second stage of labor, [hh:mm], median (IQR)	01:26 (00:40–02:25)	00:18 (00:09–00:40)	** < 0.001**	**0.26** **(0.24–0.27)**
Placental stage [hh:mm], median (IQR)	00:09 (00:06–00:14)	00:09 (00:06–00:13)	0.06	1.0 (1.0–1.1)

Bold significance was p < 0.05

Analyzing the consecutive deliveries, the active first and second stage of labor were 26 and 33% shorter in the consecutive birth when comparing the second (P1) and the third (P2) delivery (median 0.74 and 0.67, *p* < 0.001) (Table 3).

**Table 3:** Duration of different stages of labour in primiparous (P1) and women that gave birth twice before (P2). Values are presented in hours and minutes (hh:mm), median and interquartile range (IQR) as well as the ratio of the medians with a confidential interval (CI) of 95%

	P1 *N* = 2987	P2 *N* = 1176	*p*-value	Ratio P2:P1 (95% CI)
Active first stage of labour, [hh:mm], median (IQR)	02:25 (01:15–04:05)	02:05 (01:01–03:40)	** < 0.001**	**0.74 (0.69–0.82)**
Second stage of labour, [hh:mm], median (IQR)	00:18 (00:09–00:40)	00:12 (00:07–00:27)	** < 0.001**	**0.67 (0.61–0.74)**
Placental stage, [hh:mm], median (IQR)	00:09 (00:06–00:13)	00:09 (00:06–00:12)	0.03	0.93 (0.88–1.0)

Bold significance was p < 0.05

These differences disappeared beyond the third delivery for the active first (*p* = 0.11) and the second stage (*p* = 0.29) of labor (Table 4).

**Table 4:** Duration of different stages of labor comparing women that gave birth twice (P2) and tree times (P3) before. Values are presented in hours and minutes (hh:mm), median and interquartile range (IQR) as well as the ratio of the medians with a confidential interval (CI) of 95%

	P2 *N* = 1176	P3 *N* = 302	*p* value	Ratio P3:P2 (95% CI)
Active first stage of labor, [hh:mm], median (IQR)	02:05 (01:01–03:40)	02:09 (01:00–03:47)	0.11	0.9 (0.75–1.03)
Second stage of labor, [hh:mm], median (IQR)	00:12 (00:07–00:27)	00:12 (00:07–00:24)	0.29	0.88 (0.79–1.00)
Placenta stage, [hh:mm], median (IQR)	00:09 (00:06–00:12)	00:08 (00:05–00:12)	0.7	1.0 (0.85–1.24)

An overview of the durations of the active first and the second stage of labor for all parities is given in Figs. 2 and 3, respectively.

### Secondary endpoints

An overview of the potential influencing factors of the duration of labor in nulliparous and multiparous women is presented in Table 5. Neonatal birthweight (*p* = 0.83) and head circumference (*p* = 0.5) did not significantly differ between children consecutively born by the same parturient, while there was a slight tendency to an increased fetal weight with rising parity. Use and form of peripartal analgesics was significantly different in the first vs. the following births. The rate of neuraxial anesthesia (*p* < 0.001) was significantly higher in nulliparous women compared to multiparous women, whereas the use of nitrous gas (*p* < 0.001) or no analgesic therapy at all (*p* < 0.001) was significantly higher in multiparous women compared to nulliparous women. Furthermore, nulliparous women had a significantly higher rate of vaginal operative deliveries compared to multiparous women (*p* < 0.001).

**Table 5:** Secondary outcomes compared between nulliparous (P0) and multiparous (P1) (P2). Values are presented in N (%) as well as in medians and interquartile range (IQR).

	P0 *N* = 2371 (91%)	P1 *N* = 2533 (85%)	P2 *N* = 941 (80%)	*p* value
Analgesia				
No analgesia	198 (8%)	673 (23%)	333 (28%)	** < 0.001**
Nitrous oxide	97 (4%)	192 (6%)	83 (7%)	** < 0.001**
PCA (i.v. remifentanil)	18 (1%)	12 (0.4%)	8 (1%)	**0.36**
Neuroaxial anesthesia	1237 (48%)	851 (28%)	252 (21%)	** < 0.001**
Induction of labor				
Yes	651 (25%)	723 (24%)	321 (27%)	**0.06**
No	1950 (75%)	2264 (76%)	855 (73%)	
Mode of delivery				
Vaginal	1825 (70%)	2951 (99%)	1155 (98%)	** < 0.001**
Vaginal operative	776 (30%)	36 (1%)	21(2%)	
Neonatal weight [gram], median (IQR)	3320 (3040–3600)	3420 (3150–3730)	3450 (3186–3780)	0.83
Neonatal head circumference [cm], median (IQR)	35 (34–36)	35 (34–36)	35 (34–36)	0.5

Bold significance was p < 0.05

NB Data about analgesia was available for a total of 2371 women for P0, 2533 women for P1 and 941 women for P2

The multivariate regression analysis revealed neuraxial anesthesia to be an independent influencing factor to prolong first and second stage of labor irrespectively of the parity (P0 vs. P1, *p* < 0.001 and *p* < 0.001/ P1 vs. P2, *p* = 0.003 and *p* = 0.005). Higher birthweight of the first child significantly decreased the rate of second stage of labor between nulliparous and primiparous women (*p* = 0.003).

No other significant correlations between rate of labor and parity were observed: neither head circumference nor other forms of anesthesia (gas, remifentanil i.v.) influenced the duration of labor.

## Discussion

### Main findings

This study shows that till the third birth, duration of labor significantly decreases with every consecutive delivery of a parturient. Comparing the second with the first birth of a same parturient, the active first stage of labor was 51% (ratio 0.49 (95% CI 0.47–0.51, *p* < 0.001)) and the second stage 74% shorter (ratio 0.26 (95% CI 0.24–0.27, *p* < 0.001)). Counseling concerning duration of labor in multiparous should thus be individualized.

The following aspects deserve a more detailed consideration:

### Duration of labor

Duration of labor is shorter in multiparous compared to nulliparous women, with a range for nulliparous (0.29–1.57 cm/h), and multiparous (0.32–4.47 cm/h) women [4–6]. However, comparability between studies is compromised as the definition of the onset of the active first stage of labor differs. Suggested infliction points of the active first stage vary from 4 cm, 5–5.5 cm to 6–7 cm [4, 5, 8]. A large meta-analysis analyzing the duration of labor in ‘low-risk’ women found that median duration of the active first stage (beginning at 4 cm cervical dilatation, thus using the same definition as we did) was 4–8 h in nulliparous women with the upper limits of up to 20 h and 2–5 h in multiparous women up to 14 h [8]. The second stage was often completed within the first hour in nulliparous women but could last up to 4 h with neuraxial anesthesia [8]. Multiparous women completed the second stage often within 30 min but could also take up to 2 h with neuraxial anesthesia [8]. Our results are in line with these suggested times: the active first stage in nulliparous women was 2 h 50 min (lower quartile) to 7 h 40 min (upper quartile) and 1 h 15 min to 4 h 5 min in primiparous women. The median time in which the second stage was completed was 1 h 26 min for nulli- and 12 to 18 min for multiparous women.

Of note, all these above cited studies compare a cohort of nulliparous with a cohort of multiparous women, thus, not allowing an individualized interpretation of the duration of labor between first, second, and consecutive further deliveries. In general, the duration of labor varies from one woman to another. Clearly, labor progresses much faster in multiparous women. However, the above considerations and especially results of our study emphasize on an individualized look at the estimation of the length of labor and should encourage clinicians to tolerate slower progressing labors in multiparous women that experienced rather long first births, provided maternal and fetal conditions are reassuring.

We could also show that beyond the third delivery, labor progression did not get significantly faster in the following deliveries. Interestingly, a similar observation was made by Guedalia et al. [9], who showed that partograms do not differ if multiparas deliver 2–5 or 6 and more consecutive children. This observation suggests that there seems to be a certain time frame for labor that cannot get any shorter.

### Potential influencing factors of the duration of labor

Neuraxial anesthesia is known to slow down birth progression [9–12]. Consequently, longer durations of the second stage of labor are accepted in women with compared to women without neuraxial anesthesia (3 vs. 2 h in nulliparous and 2 vs. 1 h in multiparous women) [13]. Our study confirms these results, showing that neuraxial anesthesia independently tends to slower birth rates, irrespectively from parity.

Cheng et al. [14] described several elements, such as fetal size and position, to impact the duration of the second stage of labor. Further, a larger fetal head circumference was shown to lead to a prolonged second stage of labor, independently from parity or neuraxial anesthesia [15]. Additionally, sonographically measured biparietal diameter of the fetus was associated with the duration of the second stage of labor [16].

In our study, there was no significant difference between the head circumference and the birthweight between nulli- and multiparous women when comparing the two groups. However, higher birthweight seems to influence the rate of second stage of labor from the first to the subsequent pregnancy. Head circumference did not influence the rate of second stage of labor from the first to any following pregnancy.

Again, this information can be helpful for an individualized counseling of the ongoing parents about the length of labor and can potentially reduce unnecessary concerns of multiparous women.

### Limitations and strengths

One limitation is the lack of information about the latent phase as our database was not complete enough to allow a reliable and reproducible analysis of this stage of labor. Further, interpretation of influencing factors, especially the role of the neuraxial analgesia is limited to a certain point as data are not complete.

However, this study compares duration of delivery in a large cohort of women with consecutive deliveries in a single center with standardized protocols. Data were collected routinely by the involved clinicians (midwifes and obstetricians), and start and end points of the different stages of labor were clearly defined and identical for all women included in this study. Further, this study emphasizes personalized medicine, encouraging an individualized look at the estimation of the length of labor regarding the prior history of multiparous women.

## Conclusion

Till the third birth, duration of labor significantly decreases with each consecutive delivery of one parturient. The active first stage is 50%, and the second stage 75% shorter in the second compared to the first birth. Neuraxial anesthesia was found to be an independent factor influencing the duration of labor, regardless from parity. While neither birthweight nor head circumference was significantly different between children consecutively born by the same parturient, but higher birthweight of the first child significantly shortened the second stage of labor in primiparous women.

An individualized look at the estimation of the length of labor in multiparous women should be encouraged.

## Data Availability

The data that support the findings of this study are available on request from the corresponding author, Jessica Kreienbühl. The data are not publicly available due to containing information that could compromise the privacy of research participants.
